# AI-driven risk scores: should social scoring and polygenic scores based on ethnicity be equally prohibited?

**DOI:** 10.3389/fgene.2023.1169580

**Published:** 2023-05-30

**Authors:** Aviad Raz, Jusaku Minari

**Affiliations:** ^1^ Department of Sociology and Anthropology, Ben-Gurion University of the Negev, Beer-Sheba, Israel; ^2^Uehiro Research Division for iPS Cell Ethics, Center for iPS Cell Research and Application (CiRA), Kyoto University, Kyoto, Japan

**Keywords:** polygenic risk score (PRS), artificial intelligence, stratificati on, ethnicity, transparency

## Introduction

Big data, juxtaposing genetic, clinical, and socio-demographic information, forms the basis for research on various health risk correlations in precision/personalized medicine. In this context, artificial intelligence (AI) has recently been used to improve polygenic risk score (PRS). Polygenic risk scores provide a measure of individual disease risk based on one’s genome-wide information, with a particular focus on a statistical calculation of multiple genomic variants^1^. The development of population-level genetic studies, such as genome wide association studies (GWAS), has accelerated the development of PRS as part of genomic research. This characteristic, where PRS is based on a particular population, leads to an inherent need to avoid overfitting and underfitting and to address diversity in the development of the scores. Previous studies comparing PRS predictive accuracy for biobank data from different countries have shown that genetic prediction accuracy (based on UK biobank data) was far lower in non-European populations. Indeed, it was 2.5-fold lower in East Asians and 4.9-fold lower in Africans, on average (Martin et al., 2019). This poorer predictive power of PRS in non-European populations, particularly among African ancestry individuals, is most likely due to them being underrepresented within the training data. In the same vein, PRS for breast cancer in African American women based largely on variants identified in European-ancestry populations show poor performance, as DNA susceptibility loci are not similar across race/ethnicity, and have indeed been shown to differ most often for individuals of African ancestry because of their considerably greater genetic diversity (Feng et al., 2017). The way in which each individual variant affects the polygenic score can vary from study to study, adding to the complexity. In addition, using AI for PRS increases the complexity of ethical and social challenges, especially when electronic health records are integrated (Fritzsche et al., 2023). While research on PRS is ongoing, its clinical validity is still debated (Slunecka et al., 2021). Nevertheless, commercial genomic sequencing laboratories are already offering an array of both clinical and direct-to-consumer tests that include PRS as part of their risk prediction products for a variety of diseases and conditions (James et al., 2021).

We wish to draw attention to the importance and timeliness of comparing some key issues that are more broadly emerging from the proposed EU AI Act, especially regarding the banning of “social scoring” in AI systems, with the ethical concerns related to PRS. In particular, when used as a form of ethnicity-related genomic scoring, PRS with poorer predictive power in underrepresented populations could exacerbate ethnically based health discrimination as well as reinforce a reckoning with the relevance of self-reported race, ethnicity, and ancestry, and the relationship of such biomarkers and risk factors to disease diagnoses. PRS is geared primarily toward healthcare/medicine whereas social scoring is used in various areas (e.g., education, finance, insurances, migration, etc.), as well as in healthcare/medicine. While PRS are also developed for other areas than healthcare, e.g., for educational purposes (Merz et al., 2022), such educational attainment polygenic scores are similarly vulnerable to biases due to stratification, thus again highlighting the need for the critical reflection raised in this opinion. While PRS is not the same as social scoring, highlighting the differences and similarities will open up the interface of AI and health risk construction to an even wider audience.

### Criticism of PRS in the context of ethnic/ancestry traits

There is no well-established *genetic* basis for distinctly stratifying human populations by ethnicity (Mersha and Beck, 2020). However, adding parameters of ethnicity to the calculation of polygenic risk scores may reveal statistical correlations and thus interest researchers. It is now widely accepted that most of the genetic diversity in the human species exists between individuals within populations and that only a small fraction of the total genetic diversity is related to variation between ethnic populations (Kaplan & Fullerton, 2022). As geneticist Richard Lewontin (1972) famously asserted, these features of human genetic variation mean that racial classification is of “virtually no genetic or taxonomic significance” and hence should be abandoned. Recently, there are calls for building genetic literacy through education that uses population thinking and multifactorial genetics to refute genetic essentialist beliefs about race (Little et al., 2022). However, with PRS targeting “risk groups,” we are currently witnessing the resurfacing of traditional social groupings like ethnicity and race, re-charged by genomic designations. When risk estimates are applied to patients stratified by self-identified race and/or ethnicity, it may result in a range of consequences, despite the often-unprecise designation of “ethnicity” and its confluence with ancestry (James et al., 2021). Clinical use of PRS could exacerbate race-based health disparities and reinforce systemic biases of self-reported race, ethnicity, and ancestry as biomarkers and risk factors to disease diagnoses (Lewis and Green, 2021). While many common complex traits and diseases differ in their prevalence between racial and/or ethnic groups, particularly in the United States, this has been shown to be the result of pronounced racial and ethnic health disparities rather than genetic differences (Yearby et al., 2022). These concerns regarding the social/ethnic aspects of PRS echo recent concerns about AI-driven social scoring.

### Criticism of AI-driven social scoring

The proposed EU AI Act (2021) explicitly bans AI system use by public authorities (expected to later include also the private sectors) for social scoring purposes. Social scoring in this context means using an AI system to evaluate the trustworthiness of individuals based on their behaviors or personal characteristics, leading to stratified treatment of individuals. Adherence to public health measures can affect social scoring, for example, following quarantine measures or receiving vaccinations (Meszaros et al., 2022). The proposed Act explains this as follows:

AI systems providing social scoring of natural persons for general purpose by public authorities or on their behalf may lead to discriminatory outcomes and the exclusion of certain groups. They may violate the right to dignity and non-discrimination and the values of equality and justice. Such AI systems evaluate or classify the trustworthiness of natural persons based on their social behaviour in multiple contexts or known or predicted personal or personality characteristics. The social score obtained from such AI systems may lead to the detrimental or unfavourable treatment of natural persons or whole groups thereof in social contexts, which are unrelated to the context in which the data was originally generated or collected or to a detrimental treatment that is disproportionate or unjustified to the gravity of their social behaviour. Such AI systems should be therefore prohibited. (EU, 2021, article 17, p. 21).

The proposed AI Act lists high-risk AI systems in areas that include, for example, biometric identification and categorization of natural persons, law enforcement, as well as migration, asylum, and border control management. The China social credit system, which allegedly rates individuals based on the aggregation and analysis of data concerning their past behaviors, would be banned by the EU Act, if it indeed uses social scoring.

## Discussion

Polygenic risk scores (PRS) and social scoring are two different concepts. PRS are used primarily in medical research and do not involve any evaluation of an individual’s behavior or personal characteristics, but rather are based solely on genetic data. Social scoring, on the other hand, refers to a system of evaluating individuals based on various social and behavioral factors, such as their credit score, online activity, criminal record, or other personal data. However, both may reproduce biases. The concerns raised here could be used to develop a critique of how AI for genomic risk stratification in healthcare/medicine should not only be regulated for representativeness of human diversity but perhaps also for potential amplification of social scoring. This is especially important, as there may be a risk of drawing conclusions from PRS about causal relationships too quickly and with insufficient knowledge of statistics and causality/correlation claims (Fritzsche et al., 2023). By lowering the statistical standards for regarding a marker as trait-associated, weighting associations by estimated effect sizes, and aggregating associations over a larger number of variants, predictive accuracy may be increased at the expense of explainability, as any clear etiological link between specific genetic changes and the phenotype of interest is obscured.

By banning social scoring as an unacceptable risk, the proposed AI Act aims to go beyond the technical robustness, privacy, and safety required by the General Data Protection Regulation (GDPR) to prevent or minimize the probability of unintentional harm in processing personal data by AI systems. The AI Act does not directly mention AI-driven PRS. Nevertheless, in addition to specifying several unacceptable risks, it establishes the goal of minimizing the risk of erroneous or biased AI-assisted decisions in critical areas, including healthcare. We must hence carefully consider PRS in the light of minimizing the risk of erroneous or biased AI-assisted decisions. Arguably, there are three major foci in the proposed AI Act that are relevant to both social scoring and polygenic scores: transparency, non-discrimination, and accountability.

(1) Transparency: The “right to explanation” formulated in the GDPR and the proposed EU AI Act require that AI systems be explainable for high-risk decision making (EU, 2021). The “black box” conundrum is manifested in the context of scoring through the non-explainable relationships between individual genomic variants, PRS and diseases phenotype, similar to the relationships between individual “accountability”, obtained/accessible personal data, and social scoring.
(2) Non-discrimination: AI systems must collect diverse data to avoid bias and prevent the uncertain decision-making and unjust use of such data toward different populations. This requirement is critical in the case of ethnicity-based PRS due to careful consideration of the diversity of the ethnicity.
(3) Accountability: Certain actors, such as the government, health maintenance organizations, or health insurance companies, should be held responsible for the unintended consequences of individual’s actions using PRS. For example, who is responsible if a PRS-based model for breast cancer screening leads to precluding a patient from accessing screening, or has the responsibilities of harm by improper screening due to risk scores that are wrongfully produced based on race and ethnicity?

Social scoring is used in various areas as well as in healthcare/medicine, but for the sake of comparison we focus here on its use in healthcare/medicine, which is the primary area of PRS. If AI-derived PRS evaluates or classifies the risk of natural persons based on their ethnic/racial self-designation (or practitioner-designated), it would be akin to AI-derived social scoring based on previous social behaviours in multiple contexts or known or predicted personal or personality characteristics. The ethnicity related PRS obtained from such AI systems may therefore lead to the detrimental or unfavourable treatment of natural persons or whole groups of persons in healthcare contexts. Further, if the model of PRS-based screening is adopted as standard clinical practice, and if risk scores are produced based on race and ethnicity, it could lead to under- or over-screening. The purpose and implications of the classification must be clear to both those making the classification and those being classified. Social scoring can be wrong due to being based on previous behaviours that are unrelated to the context of scoring or to a detrimental treatment. Ethnicity-related PRS can be wrong because of being based on ethnic/ancestry traits that are similarly unrelated to the context of scoring or to a detrimental treatment. In this case, both AI systems should thus be equally prohibited.

## Footnotes

1
https://www.genome.gov/genetics-glossary/Polygenic-Risk-Score.
